# Quality of Life of Women with Breast Cancer and Socio-Demographic Factors

**DOI:** 10.31557/APJCP.2020.21.1.185

**Published:** 2020

**Authors:** Magdalena Konieczny, Elżbieta Cipora, Katarzyna Sygit, Andrzej Fal

**Affiliations:** 1 *Jan Grodek State University in Sanok, Medical Institute, Mickiewicza 21, 38-500 Sanok, *; 2 *The President Stanisław Wojciechowski State University of Applied Sciences in Kalisz, Nowy Świat 4, 62-800 Kalisz, *; 3 *Polish Public Health Association, Bartla 5, 51-618 Wrocław, Poland. *

**Keywords:** Breast cancer, quality of life, socio-demographic factors

## Abstract

**Background.:**

Breast cancer treatment is an aggressive therapy that affects the deterioration of women’s quality of life (QOL) in many areas. Knowledge about factors that influence the assessment of the QOL is of particular importance. The aim of the study was to analyse areas of the quality of life of women with breast cancer, taking into account social and demographic factors.

**Methods:**

The research was carried included 324 women with breast cancer. The research was carried out using a diagnostic survey, the author’s questionnaire and a standardized questionnaire for measuring the QOL of women treated for breast cancer, ie the European Organization for Research and Treatment of Cancer, Quality of Life Questionnaire (EORTC) QLQ-C30 and QLQ-QLQ module BR23. Statistical analysis uses Statistica 10.0 software. The results were considered statistically significant when the calculated probability met the inequality of p <0.05.

**Results:**

A lower QoL was found in women with diagnosed breast cancer. With age, the QoL score of respondents in the area of physical, sexual and hair loss decreased, and the following symptoms intensified: pain, insomnia, lack of appetite and shoulder ailments. The age of the respondents positively correlated with the image of their own body and social functioning. Higher QOL (higher values of functional scales and lower intensity of symptoms) were found in patients in relationships (in the scope of: cognitive functioning, future prospects), with higher education (in the scope of: physical, emotional, cognitive and sexual functioning) and declaring very good situation financial (in the scope of: physical, emotional, cognitive functioning, performing social roles).

**Conclusions:**

Age, marital status, education and financial situation influenced the QOL of women with breast cancer. In the care of women with breast cancer, attention should be paid to the individualisation of the therapeutic process, with particular emphasis on psychotherapy and support by social services.

## Introduction

On a global scale, the incidence of breast cancer is assessed as one of the most common cancer diseases of women. The most current data indicate 1,700,000 new diagnoses of breast cancer per year. About 25% of oncological diagnoses among women are breast cancer (Ginsburg et al., 2017; Ferlay et al., 2015). In the world over the last 25 years, an increase of around 30% in the incidence rates in both developed and developing countries has been found (Harbeck and Gnant, 2017; Siegel et al., 2016).

Breast cancer is also a major threat to women in Poland. The incidence of this type of cancer has more than doubled during the last 30 years, and currently nearly 20.000 new cases of breast cancer are diagnosed annually in Poland (Didkowska and Wojciechowska, 2013). The diagnosis of breast cancer is associated with the need to implement long-term and aggravating treatment. Most often it includes surgery, chemotherapy, radiotherapy and causes side effects - transient or permanent, i.e. pain, fibrosis of tissues or limitation of physical activity. Oncology therapy is usually aggressive and causes fear in patients due to uncertainty about the effects of treatment and side effects. Women are afraid of disability and death, as well as the breakdown of the family. They are also often accompanied by anger, aggression, depressed mood and a sense of self-esteem (Kyranou et al., 2013). After surgical treatment due to high trauma and mental anxiety caused by total or partial loss of breasts, a “half-body / body complex” and self-esteem can occur, especially in the social aspect (Fontes et al., 2018).

Currently, medicine assumes a holistic approach to therapy, which takes into account the treatment of not only the body, but also the psyche, and the goal of treatment is to extend life as well as improve QOL (Menen, 2016). The assessment of the mental condition, emotional state of women and the acceptance of new health conditions by them can be made through QOL examination. In the face of alarming statistics and a significant increase in the number of women with breast cancer, knowledge about determinants affecting the QOL of women with this cancer is becoming more and more important. Their thorough knowledge and analysis can be used to take action to ensure the best possible comfort of life during illness. The results of such tests may also be used in the selection of appropriate methods of treatment and rehabilitation of patients (Pinto et al., 2011). Including the QOL assessment for all medical care positively affects the relationship between the patient and the doctor and strengthens the effectiveness of the therapy (Ahn, 2007). The results of these studies are very important because contemporary oncology is focused on getting the greatest chance for cure or long-term survival, while taking into account high QOL (Visser et al., 2006). A limited number of studies considering the relationship between QOL and socio-demographic factors of patients results in low individualization of the therapeutic process. Currently, the QOL assessment is treated as one of the endpoints of clinical trials. Due to the continuous progress of medicine and the frequently changing understanding of psychological mechanisms affecting QOL, such research should be constantly conducted.

The aim of the study was to analyse areas of the quality of life of women with breast cancer, taking into account social and demographic factors and their potential influence on the QoL.

## Materials and Methods

*Place of research, characteristics of the studied population, the process of data collection*

The research was carried out in Father B. Markiewicz Podkarpackie Oncological Centre in Brzozów (Poland). The study included a group of 350 women with histopathologically confirmed breast cancer. Due to the lack of questionnaires in the questionnaire, 324 correctly completed and complete research tools were accepted for analysis. Before the examination, all patients were informed about their purpose, assured about confidentiality, anonymity and voluntary participation in the research. In addition, the examined women were informed about the possibility of resigning from the study at every stage. The study included women who had histopathologically confirmed diagnosis of breast cancer, agreed to participate in the study and were treated at Father B. Markiewicz Podkarpackie Oncological Centre in Brzozów. Criteria disqualifying a patient to participate in the study were the following factors: the occurrence of cancer other than breast cancer in the last 5 years, palliative treatment and the patient’s lack of consent for participation in the study. The consent of the director of the Father B. Markiewicz Podkarpackie Oncological Centre in Brzozów. The project also received a positive opinion of the Bioethic Committee.

*Research tools*

The research was carried out by means of a diagnostic survey using the author’s questionnaire and a standardized questionnaire for measuring the quality of life of women treated for breast cancer, ie. European Organization for Research and Treatment of Cancer, Quality of Life Questionnaire (EORTC) QLQ-C30 (quality of life questionnaire) and the QLQ-BR23 module (breast cancer) (Aaronson et al., 1993). The EORTC approval was obtained for the use of the QLQ-C30 and QLQ-BR23 questionnaires. In Poland, an assessment of the validity and reliability of the QLQ-C30 questionnaire and its version of BR 23 was carried out, which confirmed the validity of their use during QOL assessment of breast cancer patients (Zawisza et al., 2007). This fact was decisive when choosing a standardized research tool. The QLQ-C30 questionnaire is used to determine QOL and the sense of (health) state, assess the functioning in the physical, emotional and social dimensions of people with a novel. The QLQ-C30 survey consists of a general scale assessing the state of health and quality of life, 5 functional scales, 3 symptomatic scales and 6 single points (questions) also determining the intensity of symptoms. There are 30 questions in the survey. The answers to most of them are defined on a 4-point scale, ie at all (1), a little (2), significantly (3), very (4), which assess the severity of the analyzed parameters. In the last two questions (numbers 29, 30), the patients assessed their health and general QOL on a scale from 1-7 (1 - very bad / bad, 7 - excellent / perfect). The EORTC QLQ-BR23 scale is used to determine the quality of life of women with breast cancer and is a complementary module for the QLQ-C30. The questionnaire contains 23 questions to be answered in a 4-step scale, i.e., at all (1), a little (2), significantly (3), very (4). For each question, the patient chose one answer. EORTC QLQ-C30 and QLQ-BR23 questionnaires were developed statistically in accordance with the EORTC guidelines). For each patient, the raw ratio and linear transformation were calculated in order to obtain the value of the coefficient (score), whose value for both scales and individual symptoms could range from 0 to 100. In the case of functional scales, their higher coefficient corresponds to a better one ( higher) level of functioning, while the higher the ratio (score) for symptomatic scales and individual symptoms, the greater the severity of the symptom and the sick one feels worse.

*Statistical analysis*

Statistica 10.0 (StatSoft Inc., 2011) was used for statistical analysis. The basic measures of descriptive statistics were calculated: the arithmetic mean (M), the median (Me) and the standard deviation (SD). Conformity of the distribution of quantitative variables with the normal distribution was studied using the Shapiro-Wilk test. In the situation when the assumptions regarding the use of parametric methods to verify statistical hypotheses were not used, nonparametric methods were used. The following tests were used: Mann-Whitney U test, Kruskal-Wallis test (together with Dunn post-hoc test) and Spearman’s rank correlation coefficient. The significance level α = 0.05 was assumed. The results were considered statistically significant when the calculated probability p was satisfied by the inequality of p <0.05.

## Results

The average age of women enrolled in the study was 52.4 (SD = 13.7) years, with the youngest patient being 26 years old and the oldest 75 years old. The largest group were respondents above 60 years of age (29.4%), while the smallest group were those aged 20-30 (3.7%). The surveyed respondents were mostly city dwellers (54.3%). Most patients lived in cities up to 10,000 residents (28.1%) and cities up to 50,000 inhabitants (17.6%), and the smallest percentage were women living in cities over 50 thousand residents (8.6%). The study group was dominated by women living in a relationship (66.1%) and with the average (35.2%) and higher education (33.3%). Most of the surveyed women stayed at work in a state-owned factory (35.5%). Almost 40% of the surveyed women rated their financial situation as very good, while 30.6% as good, and 29.6% as bad. Among the women included in the study the most were those who had two children (32.7%), slightly fewer had one (21.3%) and three (13.3%) children, while the least four (8.0%) and more (4.9%). Almost 1/5 of respondents did not have children (Table 1).

In 71.3% (231) of the examined patients, surgical treatment was used, whereas in 112 women (34.6%) mastectomy was performed, whereas in 119 patients (36.7%) the surgery was performed. The time from surgery in more than a half of the respondents (54.1%) was over 1 to 2 years, in 22.9% - less than a year, for 12.6% women - over 3 years, and for 10.4% women this was above 2 to 3 years (Table 2).

*Quality of life and marital status*

A lowering of health status and QOL (M = 52.1 - unmarried women, M = 54.8 - women in relationships). There were statistically significant differences between the functional and symptomatic rooms QLQ-C30 and QLQ-BR23 depending on the marital status of the studied women (Table 4). Despite the fact that the general assessment of QOL in free and in a relationship was similar, the differences in the value of individual scales indicate better functioning of women in relationships. Women remaining in relationships rated the cognitive functioning higher (M = 65.2-vacant, M = 73.0 in the compound, p = 0.020). On the other hand, in women, higher intensity of vegetative and physical symptoms was observed: nausea and vomiting (M = 42.6), pain (M = 28.6), dyspnoea (M = 24.5), lack of appetite (M = 45,2), constipation (M = 19.7), as well as insomnia (M = 44.5). The unmarried women were also more strongly affected by financial problems (M = 43.9) compared to women in relationships (M = 32.4). The shown differences were confirmed statistically Table 3).

Based on the QLQ-BR23 questionnaire, the respondents rated the body image the best, while the sexual functioning was the lowest. In the case of symptoms also assessed using the QLQ-BR23 questionnaire, the women surveyed showed the discomfort caused by hair overgrowth and the side effects of systemic treatment to the highest degree. Women remaining in relationships statistically significantly higher evaluated the future perspective (M = 37.6) in comparison to free women (M = 27.6). At the same time, patients in relationships experienced greater stress due to hair loss (M = 74.2) (Table 3).

*Quality of life and education*

Another variable significantly affecting the assessment of the health status and QOL of the surveyed women was education (Table 4). The values of QLQ scales were assessed in three groups of respondents: the first - declaring vocational education, the second - with secondary and third - with higher education. A general analysis of the health status and QOL of women diagnosed with breast cancer indicated a higher score in the case of women with secondary and higher education compared to women with vocational education, but this was not statistically significant. For some functional and symptomatic scale values (QLQ-C30) statistically significant differences were found. Women with higher education higher rated physical functioning (M = 78.9), emotional (M = 63.4) and cognitive (M = 73.6) compared to those with secondary and vocational education. Performed tests of multiple comparisons indicated that in the case of the respondents’ scale with vocational education differed statistically significantly in terms of physical functioning (p = 0.001), emotional (p = 0.043) and cognitive (p = 0.021) from women with higher education. The level of education differentiated QOL in terms of the assessment of the body image, sexual satisfaction, future prospects and hair loss, as a side effect of the applied therapy. In the studied groups, the area of sexual functioning and the prospect of the future were assessed low. Sexual functioning was rated the lowest by women with vocational education (M = 15.0) and average (M = 14.6). There were statistically significant differences between the arithmetic mean values for the scale - sexual functioning in the group of women with higher education and secondary and vocational education (p = 0.000). Sexual satisfaction among women who were sexually active was assessed at an average level (Table 4).

Respondents with the lowest level of education more often indicated the nuisance of symptoms associated with the disease. Statistically significant differences were found between women with vocational education and the group of women with higher education in the arithmetic mean values calculated for the following symptoms: fatigue (p = 0.000), pain (p = 0.000), insomnia (p = 0.005) and lack of appetite (p = 0.000). In the case of other scales, the existing differences were not confirmed statistically (Table 4).

Side effects of the applied therapy (p = 0.002) and arm ailments (p = 0.029) were most seldom indicated by women with higher education, and the observed differences between arithmetic means for this scale between the group of women with higher education and occupational level achieved statistical significance. Breast ailments mostly concerned women with vocational education (the highest arithmetic mean), and the difference between this group and the group of women with secondary education was confirmed statistically (p = 0.016) (Table 5).

*Quality of life and financial situation*

In the study population, differences in functional scale and symptom scale were observed depending on the financial situation of the women surveyed (Table 5). Overall health and QOL were rated the respondents who rated their financial situation as very good (M = 59.7) and good (M = 54.9). Women in a bad financial situation assessed health status and QOL at a moderately average level (M = 45.1). The shown differences were confirmed statistically (p = 0.000). A similar relationship was demonstrated in the physical functioning of the subjects. In this respect, significant variation in scores was also found depending on the financial situation - the lowest value of the average in the group of women with bad financial situation (M = 68.5), higher in the group of respondents with good (M = 77.0) and very good (M = 77.9) financial situation. Significant differences were confirmed, statistically confirmed (p = 0.000) in the scope of performing social roles in the analyzed groups of women depending on the financial situation - the highest values in the group of women declaring very good (M = 80.1) and good (M = 73.7) financial situation, and the lowest in respondents assessing financial conditions as unsatisfactory (M = 65.6). In the analysis of emotional and cognitive functioning of women diagnosed with breast cancer, significant differences were found between respondents who were in a very good and bad financial situation (emotional functioning p = 0.000, cognitive functioning p = 0.000). Higher QOL expressed in these two scales were rated by women declaring very good financial situation. This factor did not differentiate the respondents in terms of social functioning. Surveyed women who were in poor or good financial position lower rated their sexual functioning in comparison to respondents who had a very good financial situation. Nevertheless, differences between groups were so poorly marked that no statistically significant relationships were found.

In women with poor financial situation, QOL significantly deteriorated due to worsening of the following symptoms: fatigue, nausea and vomiting, pain, breathlessness, insomnia and lack of appetite. In the case of all the above-mentioned symptoms, the differences between women with a very good financial situation and an unsatisfactory (bad) situation were confirmed statistically. Women with bad financial situation (p = 0.000) more often pointed to deeper financial problems due to illness (Table 5).

The side effects of systemic treatment, arm problems and the operated breast were less pronounced in women in a very good financial situation, and the average points were: 

M = 29.5; M = 24.5; M = 21.5. There were statistically significant differences in this aspect between women in very good and bad financial situations. The obtained results are presented in Table 5.

*Quality of life and age*

A relation was found between the general assessment of health and QOL (R = -0.19, p = 0.034) and the age of the surveyed women. With age, the QOL of the respondents decreased. There were negative and significant correlations in the case of physical functioning (R = - 0.17, p = 0.002), sexual (R = - 0.37, p = 0.000) and hair loss (R = - 0.21, p = 0.004 ). It draws attention to the clearest, with average strength correlation between age and sexual functioning. The age of respondents positively correlated with social functioning (R = 0.11, p = 0.040), body image (R = 0.22, p = 0.000) and with the occurrence of pain (R = 0.12, p = 0.033), insomnia (R = 0.11, p = 0.046), no appetite (R = 0.17, p = 0.002) and shoulder discomfort (R = 0.16, p = 0.003). Detailed results are provided in Table 6.

**Table 1:** Sociodemographic Characteristics of the Studied Group

No	Characteristics	Category	N	%
1	Age (years of age)	20–30	12	3.7
		31–40	67	20.6
		41–50	70	21.6
		51–60	80	24.7
		Over 60	95	29.4
		M (SD)	52.4 (13.7)	
		Min–Max	26–75	
2	Dwelling place	Country/Village	148	45.7
		Town of up to 10 thousand dwellers	91	28.1
		Town of up to 50 thousand dwellers	57	17.6
		Town/City of over 50 thousand dwellers	28	8.6
3	Marital status	Single	110	33.9
		In relation	214	66.1
4	Education	Basic vocational	102	31.5
		Secondary/post gymnasium	114	35.2
		Higher	108	33.3
5	Source of income	Work on your own farm	31	9.6
		Work at a state-owned factory / institution	115	35.5
		Own business	29	8.9
		Retirement	51	15.7
		Pension	66	20.4
		Other	32	9.9
6	Financial situation	Very good	129	39.8
		Good	99	30.6
		Bad	96	29.6
7	Number of children	0	64	19.8
		1	69	21.3
		2	106	32.7
		3	43	13.3
		4	26	8.0
		More than 4	16	4.9

**Table 2:** Characteristics of the Examined Group in Terms of Medical Characteristics

No	Characteristics	Category	N	%
1	Surgical treatment	Yes	231	71.3
		No	93	28.7
2	Type of an operation	Mastectomy	112	34.6
		A saving operation	119	36.7
3	Time from surgery (in years)	Less than 1 year	53	22.9
		Above up to 2 years	125	54.1
		Over 2 to 3 years	24	10.4
		Over 3 years	29	12.6
4	Chemotherapy	Yes	278	85.8
		No	46	14.2
5	Radiotherapy	Yes	94	29.0
		No	230	71.0
6	Hormone therapy	Yes	56	17.3
		No	268	82.7

**Table 3:** Women's Quality of Life Assessment - Categories Related to QLQ-C30 and BR 23 and Marital Status

Functional scales and symptoms QLQ-C30 i QLQ BR-23	p	Marital Status			
		Single		In relationship	
		M±SD	Me	M±SD	Me
n		110		214	
Health status and quality of life	0.297	52.1±21.3	50	54.8±18.9	50
Functional scales ^1^					
Physical functioning	0.184	71.8±21.6	73.3	76.4±15.8	80
Performing social roles	0,424	71.5±25.7	66.7	75.1±21.3	66.7
Emotional functioning	0.422	57.7±27.6	66.7	60.9±23.5	66.7
Cognitive functioning	0.02	65.2^a^±28.2	66.7	73.0b±23.6	83.3
Social functioning	0.637	67.7±31.3	66.7	71.0±27.2	66.7
An image of your own body	0.248	63.9±34.7	66.7	60.4±32.1	66.7
Sexual function	0.264	15.8±23.2	0	18.4±23.7	0
Sexual satisfaction	0.749	45.3±33.2	33.3	46.8±27.2	33.3
The prospect of the future	0.011	27.6^a^±32.8	0	37.6^b^±35.0	33.3
The scale of the symptoms ^2^					
Fatigue	0.221	40.5±22.2	33.3	36.0±18.8	33.3
Nausea / vomiting	0.008	42.6^a^±40.6	33.3	28.9^b^±34.6	16.7
Pain	0.018	28.6^a^±23.1	33.3	22.0^b^±18.9	16.7
Shortness of breath	0.002	24.5^a^±27.7	33.3	14.8^b^±21.8	0
Insomnia	0.034	44.5^a^±30.0	33.3	36.8^b^±26.2	33.3
Lack of appetite	0.001	45.2^a^±35.4	33.3	31.3^b^±31.0	33.3
Constipation	0.008	19.7^a^±26.4	0	12.0^b^±20.6	0
Diarrhea	0.176	13.3±21.7	0	9.3±16.3	0
Financial problems	0.004	43.9^a^±34.4	33.3	32.4^b^±30.8	33.3
Side effects of systemic treatment	0.387	33.1±21.8	28.6	30.6±21.0	28.6
Ailments related to the arm	0.119	29.3±19.7	27.8	26.2±18.9	22.2
Ailments related to the breast	0.545	25.2±18.9	25	23.8±18.9	25
Hair loss	0.005	59.2^a^±37.0	66.7	74.2^b^±33.8	100

M, arithmetic mean; Me, median; SD, standard deviation; 1, higher value means a better level of functioning and quality of life (at least 0, max. 100); 2, higher value means greater severity of symptoms (at least 0, max. 100); ^a, b^, averages marked with different letters differ statistically significantly at p <0.05.

**Table 4:** Women's Quality of Life Assessment - Categories Related to QLQ-C30 and QLQ-BR 23 and Education

Functional scales and symptoms QLQ-C30 i QLQ-BR23	p	Education					
		Vocational		Secondary		Higher	
		M±SD	Me	M±SD	Me	M±SD	Me
N		102		114		108	
Health status and quality of life	0.657	51.9±18.1	50	53.2±20.8	50	56.5±19.8	58.3
Functional scales^1^							
Physical functioning	0.001	70.3^a^±18.9	73.3	75.1±18.5	80	78.9b±15.9	80
Performing social roles	0.423	71.9±21.1	66.7	74.1±23.2	66.7	75.5±24.3	66.7
Emotional functioning	0.043	55.6^a^±27.1	66.7	60.0±23.7	66.7	63.4^b^±23.9	66.7
Cognitive functioning	0.021	65.0^a^±26.1	66.7	71.9±25.7	83.3	73.6^b^±24.2	83.3
Social functioning	0.516	71.4±30.0	83.3	69.0±29.7	66.7	69.3±26.4	66.7
An image of your own body	0.292	60.6±36.4	66.7	64.7±33.0	66.7	59.2±29.3	66.7
Sexual function	0	15.0^a.b^±25.5	0	14.6^b^±21.7	0	22.8^c^±22.7	16.7
Sexual satisfaction	0.393	43.2±31.8	33.3	50.8±26.4	33.3	43.0±28.8	33.3
The prospect of the future	0.461	28.4±35.2	0	33.0±34.5	33.3	31.2±32.0	33.3
The scale of the symptoms^2^							
Fatigue	0	43.1^a^±19.7	33.3	37.4±21.6	33.3	32.3^b^±17.3	33.3
Nausea / vomiting	0.11	38.9±39.6	33.3	34.4±37.3	16.7	27.6±34.3	16.6
Pain	0	30.4^a^±20.6	33.3	24.1±20.6	16.7	18.7^b^±19.3	16.7
Shortness of breath	0.023*	22.2±26.3	16.7	19.0±24.7	0	13.3±21.4	0
Insomnia	0.005	45.8^a^±28.1	33.3	39.8±28.7	33.3	33.0^b^±25.2	33.3
Lack of appetite	0	46.4^a^±33.6	33.3	35.7±34.0	33.3	26.6^b^±29.1	33.3
Constipation	0.664	14.02±20.7	0	17.5±28.1	0	12.0±18.5	0
Diarrhea	0.81	10.8±18.3	0	11.7±19.8	0	9.6±17.1	0
Financial problems	0.057	39.9±31.8	33.3	38.3±33.0	33.3	30.9±32.1	33.3
Side effects of systemic treatment	0.002	37.2^a^±22.3	33.3	31.5±21.8	28.6	25.9^b^±18.1	23.8
Ailments related to the arm	0.029	30.4^a^±19.5	33.3	31.5±21.8	28.6	24.0^b^±15.4	22.2
Ailments related to the breast	0.016	29.4^a^±21.8	25	21.7^b^±18.5	16.7	22.1±15.2	20.8
Hair loss	0.8921	67.2±36.9	66.7	67.7±36.2	66.7	70.8±34.1	83.3

M, arithmetic mean; Me, median; SD, standard deviation; *, no statistically significant differences were found in the post hock analysis; 1, higher value means a better level of functioning and quality of life (at least 0, max. 100); 2, higher value means greater severity of symptoms (at least 0, max. 100); ^a, b^, averages marked with different letters differ statistically significantly at p <0.05.

**Table 5:** Women's Quality of Life Assessment - Categories Related to QLQ-C30 and QLQ-BR23 and the Financial Situation

Functional scales and symptoms QLQ-C30 i QLQ BR-23	p	Financial situation					
		very good		good		bad	
		M±SD	Me	M±SD	Me	M±SD	Me
N		129		99		96	
Health status and quality of life	0	59.7^a.c^±17.1	58.3	54.9^c^±19.0	50.0	45.1^b^±20.7	45.8
Functional Scales^1^							
Physical functioning	0	77.9^a.c^±15.8	80.0	77.0^c^±16.7	80.0	68.5^b^±20.7	73.3
Performing social roles	0	80.1^a^±21.0	83.3	73.7^b.c^±20.2	66.7	65.6^c^±25.5	66.7
Emotional functioning	0	66.0^a^±19.5	66.7	58.6±27.6	66.7	52.6^b^±25.5	66.7
Cognitive functioning	0	76.9^a^±21.2	83.3	69.36±25.3	66.7	62.5^b^±28.8	66.7
Social functioning	0.094	74.7±21.7	66.7	70.5±29.5	66.7	62.7±34.4	66.7
An image of your own body	0.496	61.2±28.8	66.7	64.3±33.8	66.7	59.2±37.1	66.7
Sexual function	0.041*	20.5±23.3	16.7	14.7±21.2	0.0	16.3±25.9	0.0
Sexual satisfaction	0.088	51.2±24.0	33.3	37.0±28.2	33.3	46.0±37.2	33.3
The prospect of the future	2.537	33.6±31.9	33.3	28.6±35.0	0.0	29.9±35.4	16.7
The scale of the symptoms^2^							
Fatigue	0	34.1^a.c^±19.6	33.3	33.2^c^±17.4	33.3	46.5^b^±20.6	44.4
Nausea / vomiting	0	26.6^a.c^±32.2	16.6	26.4^c^±33.8	16.7	50.2^b^±41.7	50.0
Head pains	0	18.5^a.c^±18.2	16.7	22.2^c^±18.4	16.7	34.2^b^±22.5	33.3
shortness of breath	0	12.4^a.c^±21.3	0.0	16.2^c^±22.5	0.0	27.8^b^±27.2	33.3
Insomnia	0	32.8^a.c^±27.3	33.3	35.4^c^±23.7	33.3	52.4b±28.1	33.3
Lack of appetite	0	29.2^a.c^±31.7	33.3	21.6^c^±28.1	33.3	51.7^b^±34.8	33.3
Constipation	0.031*	12.1±22.0	0.0	12.8±20.0	0.0	19.8±26.0	0.0
Diarrhea	0.263	9.3±17.2	0.0	9.8±17.3	0.0	13.5±20.9	0.0
Financial problems	0	25.1^a.c^±27.7	33.3	35.0^c^±29.9	33.3	52.8^b^±34.4	66.7
Side effects of systemic treatment	0	29.5^a.c^±20.8	28.6	26.8^c^±19.7	23.8	38.8^b^±21.8	35.7
Ailments related to the arm	0.009	24.5^a^±19.2	22.2	26.8±17.2	22.2	31.4^b^±20.5	33.3
Ailments related to the breast	0.006	21.5^a^±17.3	16.7	23.1±19.0	25	29.3^b^±19.9	25.0
Hair loss	0.887	68.7±35.7	100.0	69.4±36.9	100	67.2±35.5	66.7

M, arithmetic mean, Me - median, SD - standard deviation; *, no statistically significant differences were found in the post hock analysis; 1, higher value means a better level of functioning and quality of life (at least 0, max. 100); 2, higher value means greater severity of symptoms (at least 0, max. 100); ^a, b, c^, mean values in different letters differ statistically significantly at p <0.05.

**Table 6:** Spearman's Rank Correlation Coefficients between QLQ-C30 and OLQ-BR23 and the Age of the Subjects

Functional and symptomatiuc scales QLQ	R Spearman	p
QLQ -C30		
Health status and quality of life	-0.19	0.034
Physical functioning	-0.17	0.002
Performing social roles	-0.05	0.325
Emotional functioning	-0.05	0.382
Cognitive functioning	-0.03	0.595
Social functioning	0.11	0.04
Fatigue	0.09	0.089
Nausea / vomiting	0.04	0.474
Pain	0.12	0.033
Shortness of breath	0.09	0.118
Insomnia	0.11	0.046
Lack of appetite	0.17	0.002
Constipation	0.03	0.597
Diarrhea	0.01	0.778
Financial problems	0.1	0.067
QLQ -BR23		
An image of your own body	0.22	0
Sexual function	-0.37	0
Sexual satisfaction	-0.14	0.175
The prospect of the future	0.03	0.549
Side effects of systemic treatment	0.06	0.248
Ailments related to the arm	0.16	0.003
Ailments related to the breast	0.09	0.108
Hair loss	-0.21	0.004

## Discussion

QOL is a subjective concept and assessed from the patient’s perspective. Oncological treatment is performed on the application of effective methods of therapy, while ensuring high QOL. Understanding the factors determining QOL of women with breast cancer can indicate the directions of activities to ensure adequate comfort of life for sick women. Among the determinants influencing QOL, socio-demographic factors change.

The study evaluated QOL of women diagnosed with breast cancer depending on the following socio-demographic factors: age, marital status, education, and financial situation. The collected data show that with age, the global assessment of health and QOL of patients decreased. This dependence also occurred in the case of physical and sexual functioning. In turn, older women had a better QOL in the field of social functioning and they rated the body image more highly. With age, the occurrence of symptoms associated with the disease, such as pain, insomnia, lack of appetite and ailments of the arm on the operated side increased. The presented research results indicate that for younger women it is particularly important to assess the body image, which seems to be more critical than in the case of older women. The attention is also paid to the different type of support that should be directed to women of all ages. Younger patients, due to low assessment of the sphere of social functioning and low assessment of their own body image, need more support in this area. However, for older women, help is needed to increase their physical and sexual functioning.

The literature presents various results regarding the relationship between QOL and the age of patients. Sio et al., (2014) stated that younger women performed better in the area of physical activity, while older patients showed a good general QOL and higher assessed the body image, which largely coincides with the results presented in this paper. Huang et al., (2017) analyzing the impact of selected socio-demographic factors on QOL among 225 women with breast cancer, stated that QOL was not age-dependent. In turn, a study by Wöckel et al., (2017) showed that the QOL of young women waiting for treatment was lower than the older ones, and the treatment applied could still have a negative effect on the quality of life. In the studies of Nancy et al., (2005), the relationship between the applied therapy (chemotherapy, surgical treatment) and QOL of women with breast cancer was examined and the socio-demographic factors were analyzed to influence the existing relationships. One of the conclusions obtained as a result of these studies was the fact that QOL of women depended not only on the type of treatment, but also on the age of the subjects and their level of education, which should be taken into account in the therapeutic process.

Determining marital status as a differentiating variable in own studies, it was shown that people in relationships achieved significantly better results in terms of cognitive functioning and assessment of future prospects and had lower intensity of symptoms, especially vegetative, associated with the disease (nausea and vomiting, pain, shortness of breath, insomnia , lack of appetite, constipation). In addition, women in relationships have had less financial problems than single people. There were no statistically significant differences in the assessment of the body image and sexual function and sexual satisfaction between unmarried women and those in relationships. Similarly, in the studies of Parker et al., (2003), it was shown that the higher QOL had married patients. Also, studies carried out by Croft et al., (2014) showed that married women who were diagnosed with breast cancer after 5 years from the diagnosis had a higher level of optimism compared to unmarried women. In the studies of Cobo-Cuenca et al., (2018) and Acil and Cavdar (2014) it was shown that women in relationships had greater life satisfaction compared to unmarried women. Demonstrating the relationship between being in a relationship with QOL and women with breast cancer indicates the importance of family support in the fight against the disease.

In our own research, education was a factor that, although it did not result in differences in the overall health and QOL scores, influenced the value of some functional and symptomatic scales. Women declaring higher education were significantly better in the physical, emotional, cognitive and sexual areas compared to women with vocational education. At the same time, in women with lower levels of education, symptoms associated with the disease, such as fatigue, pain, insomnia, lack of appetite, breast and shoulder discomfort and side effects of systemic treatment were more pronounced than in women with higher education.

Recently, the importance of educational programs for patients diagnosed with cancer has been particularly emphasized. Shahsavari (2015) found that the implementation of such programs at the stage of breast cancer diagnosis, treatment options and continuation of therapy, as well as acceptance of the disease resulted in improved QOL of patients in the four domains of functioning: physical, psychological, social and emotional.

The results of own research carried out in Father B. Markiewicz Podkarpackie Oncological Centre in Brzozów indicate that another factor that significantly influenced QOL was the financial situation of women. The very good financial situation of the respondents was associated with a higher QOL, both global and expressed in the form of individual functional and symptomatic scales. Women who declared a very good financial situation achieved higher results in terms of physical functioning, fulfilling social roles, emotional and religious functions. As a consequence, also symptoms associated with cancer were more burdensome for women with worse financial situation compared to better-off. Almost all analyzed symptomatic scales showed a stronger intensity of symptoms in the group of women with unsatisfactory financial situation. It is noteworthy that the differences shown in the majority take on a linear character. Similar results were obtained by Huang et al., (2017) and Yan et al., (2016). In their studies, they found higher QOL in breast cancer patients who had higher income. Also in the studies of Kobayashi et al., (2008), it was shown that higher family income was positively correlated with better overall health and QOL of women. In contrast, loss of job negatively correlated with all functional scales of the QLQ-C30. Similar results in their research were obtained by Jendrian et al., (2017), in which they stated that predictors of good QOL among women diagnosed with breast cancer were among others higher monthly family income and higher professional qualifications.

The results presented by Ramadas et al., (2015) indicated that QOL of women diagnosed with breast cancer was higher in those respondents who were married and lived with their family. In these studies, it was found that women who did not have children significantly worse in the psychological area compared to patients with children.

Obtained results of own research and other authors indicate the great importance of socio-demographic factors for QOL of women diagnosed with breast cancer.

## Conclusions

1. The age, marital status, education and financial situation of women diagnosed with breast cancer had an impact on their QOL.

2. In order to improve the quality of life of women, special care should be given to the elderly in the area of physical and sexual functioning and discomforts resulting from the disease, while in the group of younger women in the field of social functioning and improving the assessment of their body image.

3. Professional care that strengthens the functioning of women with breast cancer in all areas of life should be addressed especially to women who are not in relationships, with a lower level of education and in a worse financial situation.

4. A great role in caring for patients with breast cancer should be played by social monitoring and providing assistance by appropriate organizations and social services.

## References

[B1] Aaronson NK, Ahmedzai S, Bergman B (1993). The European Organisation for Research and Treatment of Cancer QLQ-C30: A quality-of-life instrument for use in international clinical trials in oncology. J Natl Cancer Inst.

[B2] Acil H, Cavdar I (2014). Comparison of quality of life of Turkish breast cancer patients receiving breast conserving surgery or-modified radical mastectomy. Asian Pac J Cancer Prev.

[B3] Ahn SH, Park BW, Noh DY (2007). Health related quality of life in disease-free survivors of breast cancer with general population. Ann Oncol.

[B4] Cobo-Cuenca AI, Martín-Espinosa NM, Rodríguez-Borrego MA (2019). Determinants of satisfaction with life and self-esteem in women with breast cancer. Qual Life Res.

[B5] Croft L, Sorkin J, Gallicchio L (2014). Marital status and optimism score among breast cancer survivors. Support Care Cancer.

[B6] Didkowska J, Wojciechowska U (2013). Breast cancer in Poland and Europe - population and statistics. Nowotwory. J Oncol.

[B7] Ferlay J, Soerjomataram I, Dikshit R (2015). Cancer incidence and mortality worldwide: sources, methods and major patterns in GLOBOCAN 2012. Int J Cancer.

[B8] Fontes KP, Veiga DF, Naldoni AC (2019). Physical activity, functional ability, and quality of life after breast cancer surgery. J Plast Reconstr Aesthet Surg.

[B9] Ginsburg O, Bray F, Coleman MP (2017). The global burden of women’s cancers: a grand challenge in global health. Lancet.

[B10] Harbeck N, Gnant M (2017). Breast cancer. Lancet.

[B11] Huang HY, Tsai WC, Chou WY (2017). Quality of life of breast and cervical cancer survivors. BMC Womens Health.

[B12] Jendrian S, Steffens K, Schmalfeldt B (2017). Quality of life in patients with recurrent breast cancer after second breast-conserving therapy in comparison with mastectomy: the German experience. Breast Cancer Res Treat.

[B13] Kobayashi K, Morita S, Shimonagayoshi M, el al (2008). Effects of socioeconomic factors and cancer survivors’ worries on their quality of life (QOL) in Japan. Psychooncology.

[B14] Kyranou M, Paul SM, Dunn LB (2013). Differences in depression, anxiety, and quality of life between women with and without breast pain prior to breast cancer surgery. Eur J Oncol Nurs.

[B15] Menen RS, Hunt KK (2016). Considerations for the treatment of young patients with breast cancer. Breast J.

[B16] Parker PA, Baile WF, De Moor C, Cohen L (2003). Psychosocial and demographic predictors of quality of life in a large sample of cancer patients. Psychooncology.

[B17] Pinto AC, de Azambuja E (2011). Improving quality of life after breast cancer: Dealing with symptoms. Maturitas.

[B18] Ramadas A, Qureshi AM, Dominic NA (2015). Socio-demography and medical history as predictors of health-related quality of life of breast cancer survivors. Asian Pac J Cancer Prev.

[B19] Shahsavari H, Matory P, Zare Z, Taleghani F, Kaji MA (2015). Effect of self-care education on the quality of life in patients with breast cance. J Educ Health Promot.

[B20] Siegel RL, Miller KD, Jemal A (2016). Cancer statistics, 2016. CA Cancer J Clin.

[B21] Sio TT, Chang K, Jayakrishnan R (2013). Patient age is related to decision-making, treatment selection, and perceived quality of life in breast cancer survivors. World J Surg Oncol.

[B22] Visser MR, van Lanschot JJ, van der Velden J (2006). Quality of life in newly diagnosed cancer patients waiting for surgery is seriously impaired. J Surg Oncol.

[B23] Wöckel A, Schwentner L, Krockenberger M (2017). Predictors of the course of quality of life during therapy in women with primary breast cancer. Qual Life Res.

[B24] Yan B, Yang L-M, Hao L-P (2016). Determinants of Quality of Life for Breast Cancer Patients in Shanghai, China. PLoS One.

[B25] Zawisza K, Tobiasz-Adamczyk B, Nowak W (2010). Validity and reliability of the quality of life questionnaire (EORTC QLQ C30) and its breast cancer module (EORTC QLQ BR23). Ginekol Pol.

